# Local Norms and the Theory of Planned Behavior: Understanding the Effects of Spatial Proximity on Recycling Intentions and Self-Reported Behavior

**DOI:** 10.3389/fpsyg.2019.00744

**Published:** 2019-03-29

**Authors:** Paola Passafaro, Stefano Livi, Ankica Kosic

**Affiliations:** Department of Developmental and Social Psychology, Sapienza University of Rome, Rome, Italy

**Keywords:** local norms, theory of planned behavior, social norms, descriptive norms, spatial proximity, recycling, environmental behavior

## Abstract

This paper aims to deepen the understanding of the role of “local norms” in explaining ecological behavior within Ajzen’s Theory of Planned Behavior. A longitudinal investigation (overall *N* = 222), focused on households waste recycling, tested the hypothesis that the effects of this type of norms on behavioral intentions varies as a function of the individual’s spatial proximity to the social categories relevant to the social-physical context (in this study: housemates, neighbors, inhabitants of the district or quarter, and inhabitants of the city) in which the behavior takes place. The hypothesis was confirmed and we also showed that the effects of local norms are empirically distinguishable from those of the social norms already considered by the model (i.e., subjective norms). Local norms, also have a direct influence on self-reported recycling behavior measured 1 month after intentions. We propose possible theoretical explanations for the results obtained and discuss the implications for applicative purposes.

## Introduction

Normative influence has become a key component of various social-psychological models of behavioral decision making, including the Theory of Planned Behavior (TPB; e.g., Ajzen, 1991, 2012; Fishbein and Ajzen, 2010). Although the TPB has consistently been shown to be overall valid and effective (e.g., Ajzen, 2015), the relative importance of its components in the prediction of intention is “expected to vary across behaviors and situations” (Ajzen, 1991, pg. 189). In particular, attitudes and perceived behavioral control (PBC) tend to show stable, consistent effects on intentions, whereas the results relating to subjective norms have always been mixed (Ajzen, 1991; Trafimow and Finlay, 1996; Armitage and Conner, 2001). According to Ajzen (1991, 2015) the inconsistency should mostly be attributable to measurement problems linked to the construct validity and reliability of the measures used in specific studies. However, some authors have attributed the inconsistent results to the existence of different kinds of social norms, not all of which are captured by the ways in which the subjective norms construct is typically operationalized within the TPB. In particular, Fornara et al. (2011) have shown that indicators of subjective norms may fail to capture the normative influence derived from people sharing a same spatial-physical setting. They have suggested that norms of this kind, termed “local norms," are relevant to behaviors that have collective, spatially defined implications, such as household waste recycling. Some studies have confirmed that “local norms" can explain additional proportion of variance in behavior, over and above that explained by the original components of the TPB (see also Carrus et al., 2009). Nevertheless, the role of local norms within the TPB framework is far from being fully understood. Amongst the issues that remain unclear is whether the effects of local norms on intentions vary as a function of the level of spatial proximity, and how much direct influence they have on self-reported behavior. The study reported here intend to provide some empirical evidence of the relevance of these aspects to particular types of ecological behavior studied within the framework of the TPB model.

### Normative Influence and the TPB

The TPB (e.g., Ajzen, 1991) is an established, social psychological model of human behavior with a relatively parsimonious structure based on five key factors: attitudes, subjective norms, PBC, intentions, and behavior. Attitude, subjective norms, and perceived behavior control are assumed to influence behavioral intentions, which, in turn, are assumed to affect behavior more directly. The model also posits, that, in some cases, PBC may have a direct effect on behavior. The theory has been used successfully in hundreds of studies and various disciplinary domains to understand and predict an impressive variety of individual behaviors (e.g., Ajzen, 2012, 2015), including taking vitamins (e.g., Madden et al., 1992), doing leisure activities (e.g., Pierro et al., 2003), attending academic classes and obtaining the corresponding achievements (e.g., Ajzen and Madden, 1986), following medical prescriptions (e.g., Livi et al., 2017), watching one’s own weight (Bagozzi and Kimmel, 1995), orienting consumer behavior (e.g., Mannetti et al., 2002), as well as engaging in ecological behaviors (for a review of early studies see e.g., Staats, 2003; for examples of investigations, see Terry et al., 1999; Mannetti et al., 2004; Nigbur et al., 2010; Chan and Bishop, 2013). However, it seems not possible to determine the relative weight of the various original components *a priori* and this represents the most variable aspect of the model. In many cases this variability can be attributed to measurement issues (i.e., when the construction of items does not follow the original indications of the authors; e.g., Fishbein and Ajzen, 2010; Ajzen, 2015), but there are situations in which the problem seems to arise from the model’s lack of contextualization. Such contextualization, although repeatedly recommended, has mostly been associated with assessment of the specific behavioral, normative and control beliefs relevant to a given context (for a recent example see, e.g., De Leeuw et al., 2015). The author of the model supports the idea that assessing specific beliefs associated with a given behavior is crucial to the practical application of the model (e.g., Bamberg and Schmidt, 2001; Livi et al., 2017), but the theoretical importance of beliefs in explaining intentions has been often neglected. In fact, the model implies that specific beliefs do not have a direct effect on intentions as this is assumed to be fully mediated by the main general components of the theory (i.e., attitude, subjective norms, and PBC). Regarding social norms, in particular, the model assumes that the construct of subjective norms is sufficient to account for the effects of all the specific social groups or categories relevant to a particular behavior in a particular context, and that there is no room for specific groups related effects to explain additional variance in intentions directly (a study supporting this view is reported in Bodimeade et al., 2014). However, there are reports of cases in which additional variance has been explained by measures of normative influence referred to specific social (referent) groups or categories (e.g., Sheeran and Orbell, 1999; Fekadu and Kraft, 2002; Louis et al., 2007; White et al., 2009). Moreover, factor analyses have shown that measures of beliefs theoretically associated with different kinds of social norms can saturate on separate factors (Grube et al., 1986; Sheeran and Orbell, 1999; White et al., 1994). Some authors have suggested that this could be due to the way in which subjective norms are typically operationalized within the TPB, that is by asking people to indicate their level of agreement with statements such as “people important to me would approve/not approve if I …...” According to Terry et al. (2000), referring to generic “people important to me” can leave respondents uncertain about exactly who is being referred to, which may, in some cases, weaken the overall predictive power of norms measured in this way. Another possibility, however, is that, this question format – the most used formulation for measuring subjective norms – could lead respondents to refer, if not to a well- defined group, at least to a well- defined range of people. In particular, it could lead most respondents to refer to people who are affectively and positively important for them, i.e., people with whom they share close and personally meaningful relationships (e.g., Vesely and Klöckner, 2018). These people are typically family members, partners and close friends, who play a primary role in an individual’s behavioral decisions in many domains, including those where the TPB has been widely tested and received the greatest support (like, for example, the health domain). Nevertheless, there may be domains and contexts in which the relevance of this kind of people is lower, leaving more room for the effects of other types of normative influence.

### Distinguishing the Sources of Normative Influence on Ecological Behavior

The literature on social influence suggests that, when conforming to social norms, individuals do not always (or exclusively) refer to close friends, relatives and partners. There are situations in which one can also decide to behave similarly to people with whom one does not share strong affective bonds or consolidated social relationships. This fact was first demonstrated empirically by Asch’s (e.g., Asch, 1955, 1956) classical experiments on conformity in small groups which showed that individuals sometimes altered their judgments to conform to those of people they had never met before. Cialdini et al. (1990, 1991) field experiments on littering have shown that this can happen even when no one other is physically present in the context in which the target behavior occurs. This because individuals are able to derive information about others’ behavior from cues in the physical environment (see also Cialdini, 2005, 2007; Goldstein et al., 2008). This means that the simple fact of sharing the same social-physical space with others, in a given moment or period of time, can be a potential source of reciprocal normative influence, even in absence of strong, direct social or affective bonds. Fornara et al. (2011) noticed, however, that classic models of social behavior such as the TPB have always tended to underestimate this type of normative influence (see also Carrus et al., 2009). Models like the TPB have mostly focused their attention on more “de-localized” conceptions of social norms (represented by subjective norms) and by either personal social norms (when adopting an individual perspective) or group norms (when adopting a group perspective). Fornara et al. (2011) argued that, whilst these kinds of normative influence proved adequate for explaining behavioral decisions with personal, interpersonal or intergroup implications, they can only partially explain behavioral decisions which also have broader, collective or territorial implications. They posited that the latter kind of behavioral decisions is also influenced by a kind of social influence derived from spatially anchored social interactions and suggested these influences should be referred to as “local norms” or “place norms.” This term was deemed particularly appropriate for the normative influence potentially originating from the association between a particular behavior and the specific social-physical territory in which that behavior occurs.

This kind of normative influence should be distinguishable from the kind described by theories such as social identity theory (SIT), which is typically moderated by the individual’s level of identification with a particular social group (e.g., Terry et al., 1999). Local norms, instead, are expected to exert a direct effect on intentions as they are supposed to derive from social-cognitive processes other than group identification. One hypothesis refers to their information potential, also known as the “adaptive” value of social norms. Cialdini et al. (1991) have argued that, by observing what others do in a certain situation, people can rapidly learn the apparently “wisest” way to behave in that specific context. Moreover, studies on social dilemmas (e.g., Van Lange et al., 2013) have suggested that when the overall effectiveness of individual behavior depends on the collective participation of others, it is likely that the individual’s perception of others’ behavior will influence their perception of how worthwhile their personal efforts would be. A study of water consumption by Corral-Verdugo et al. (2002) is particularly pertinent to this issue. These authors noticed that individuals were more motivated to reduce water consumption if they perceived that other people living in the same residential area were also trying to reduce theirs. Hence, by observing the behaviors of others sharing the same environment individuals can develop their own idea of the extent to which their own efforts are likely to be effective in contributing to achieve the intended collective goal. Moussaoui and Desrichard (2017) found that this perception can mediate the relationship between descriptive norms and intentions. Studies of household recycling intentions (a behavior that has clear collective goals and implications) by Fornara et al. (2011) and Carrus et al. (2009) have confirmed the important role of local norms referring to neighbors, but more research is needed to improve the understanding of the nature and functioning of this kind of norms within the TPB framework. In particular, if the sphere of influence of local norms is spatially determined, then their effects on behavioral intentions should vary with the individual’s spatial proximity to the physical space from which they derive. Some evidence of the importance of spatial proximity on individual behavior has been found in the health domain (e.g., Carroll et al., 2018). In relation to household waste recycling, it is reasonable to expect that local norms referring to people who live close to the subject would exert a greater effect on intentions than norms derived from people who are geographically more distant. This effect should, then, be distinguishable from the normative influence of people who are important to the subject (i.e., the so called subjective norms) and could be attributed to a number of factors. One is related to information accuracy. People tend to have more chances to check on the behavior of those who live nearby rather than at a further distance. Moreover, the focus theory of normative influence (Cialdini et al., 1990) posits that others’ behavior tends to have an “on-site” “reminder” function able to modulate the salience of social norms relevant for a specific context (see also Krupka and Weber, 2009). For the same reason, one might expect local norms to have a direct effect on behavior and on the intention-behavior relationship. In particular, they may act as a kind of environmental cue that can directly “trigger” a certain behavior (e.g., Chartrand and Bargh, 1999; Bargh et al., 2012). Some studies have shown how, in certain circumstances, social norms moderate the intention-behavior relationship (e.g., Bodimeade et al., 2014). However, none of these possibilities has ever been explored in previous research on local norms within the TPB framework.

### Aims and Hypothesis

The general goal of this study was to deepen the understanding of the effects of local norms on behavioral intentions within the TPB framework and to explore their possible influence on self-reported household waste recycling behavior. In particular, we wanted to assess whether the additional contribution of local norms to the TPB varied in function of the level of spatial proximity to the individual, and whether this factor represented an additional predictor of self-reported behavior, over and above behavioral intention. To this aim, we thought to explore and compare the normative influence of people living close (i.e., in the same house as the target individuals) with that of people living progressively further away (i.e., neighbors, and inhabitants of the same city). Based on previous studies, we expected to confirm that, in general (H1) the original TPB variables would significantly predict behavioral intentions to recycle household waste at Time 1, and that intentions would predict self-reported behavior at Time 2 (1 month later). Moreover, we specifically hypothesized that: (H2) Local norms would explain additional variance in intentions at Time 1, with the normative influence of people living close to respondents (housemates and neighbors) showing a greater predictive capacity (*vis a vis* behavioral intentions) compared to the normative influence of people living progressively further away (people leaving in the same city). Finally we expected that (H3) local norms would reveal to be statistically significant predictors of self-reported behavior at Time 2, in addition to behavioral intentions.

## Materials and Methods

### Research Design

We used a longitudinal, survey type study to test our hypotheses. Two different questionnaires were developed and administered to participants at two distinct moments, Time 1 and Time 2 (1 month after Time1). In earlier research on local norms, Carrus et al. (2009) and Fornara et al. (2011) used samples that were relatively heterogeneous for age and education, but we decided to recruit a sample consisting sole of university students. As well as being convenient, this was done in order to increase the representation of individuals sharing living space with people with whom they did not have strong social or affective bonds. In Italy, it is uncommon for adults to live in such circumstances, whereas it is typical of young students who have moved from small towns across the country to a university city. Many of these students rent a room in shared apartments, typically close to university facilities, and they tend not to have particular social or affective bonds with their housemates, whom they often meet on site. Moreover, many of these cohabitations are temporary, being limited to a single academic year.

#### Measurement Instrument

The measures used in the questionnaires follow, in general, Ajzen’s suggestions for measuring the TPB constructs (e.g., Fishbein and Ajzen, 2010; see also^1^) and represent an adaptation to this specific context of those used in previous research work on local norms (e.g., Carrus et al., 2009; Fornara et al., 2011). The first questionnaire (used at Time 1) was created to measure all the TPB original constructs as well as Local Norms. More specifically:

- *Subjective norms*, Particular attention was dedicated to the way in which normative influence has been implemented in this questionnaire. Previous investigations on the TPB had shown that differences in the predictive capacity of subjective norms on intentions could be linked to whether they had been measured as prescriptive or descriptive norms (e.g., Smith and Louis, 2008; Smith et al., 2012; Schultz et al., 2018). As we wanted to exclude the possibility that the additional effect of local norms on intentions could be attributed to measurement issues of this type, both subjective and local norms were measured (and considered in the analyses) taking into account their prescriptive and descriptive components. Hence, subjective norms were measured using four items, two of which assessed the prescriptive dimension: (1) “Most of the people who are important to me think that I should recycle household waste”; (2) “If I engaged in household recycling, in the next month, most people who are important to me would….” Other two items referred to the descriptive dimension: (3) Most of the people who are important to me do recycle household waste”; and (4) “How many among the people important for you do recycle household waste?” Responses were collected on a 6-point scale, labeled from 1 (totally likely) to 6 (totally unlikely) in the case of items 1 and 3, and from 1 (completely disagree) to 6 (completely agree) in the case of item 2. Responses for Item 4 were recorded on 6 point scale as well, but labeled as follows: 1 (none), 2 (very few), 3 (few), 4 (some of them), 5 (many of them), and 6 (all of them).
- *Local norms*, like subjective norms, were measured in their prescriptive and descriptive dimensions. The former (prescriptive) was measured with the statement “According to you, if you engaged in household waste recycling, in the next month, how much would the following people agree”: (1) people who live with you, (2) your neighbors, (3) people in your own city area, and (4) people in your own city. Responses were recorded on a 6 point scale ranging from 1 (completely disagree) to 6 (completely agree). The descriptive dimension of local norms was measured with two items. Item 1 “How often do you think that the following people recycle their household waste? (1) people who live with you, (2) your neighbors, (3) people in your own city area, and (4) people in your own city”. Responses were recorded on a 6 point scale labeled as follows: 1 (never), 2 (rarely), 3 (sometimes), 4 (often), 5 (almost always), and 6 (always). Item 2 corresponded to the question “In your view, how many of the following people do engage in recycling household waste? (1) people who live with you, (2) your neighbors, (3) people in your own city area, and (4) people in your own city”. Responses were recorded on a 6 point scale labeled as follows: 1 (none), 2 (very few), 3 (few), 4 (some of them), 5 (many of them), and 6 (all of them).
- *Attitude toward household waste recycling* was measured with the statement “For me recycling household waste is …” followed by six bipolar adjectives (good/bad; right/wrong; pleasant/unpleasant; useful/useless; important/non-important; appropriate/inappropriate) separated by a six point (unlabeled) response scale;
- *Perceived behavioral control* was measured with the statement “for me recycling household waste is …” followed by four bipolar adjectives (difficult/easy, simple/complicated, possible/impossible, and feasible/unfeasible), separated by a 6 point unlabeled response scale.
- *Behavioral Intentions* were measured with two items: Item (1) “During the next month, I intend to engage in household waste recycling,” followed by a 6 point response scale labeled 1 (totally likely) to 6 (totally unlikely). Item (2) corresponded to the question “How much are you decided to do it?” followed by a 6 point response scale ranging from 1 (totally undecided) 6 (totally decided).

The questionnaire included also measures of social-demographical nature (age, gender, and place of residence), respondents’ name and identification number, as well as other measures not considered for the purposes of this paper.

The second questionnaire (used at Time 2, i.e., 1 month later) was composed of the following two items measuring self-reported household waste recycling behavior: (1) “During last month, you have engaged in household waste recycling 6 (always), 5 (often), 4 (sometimes), 3 (few times), 2 (rarely), and 1 (never); (2) “During last month you have engaged in separately disposing of” 6 (all of your household waste), 5 (most of your household waste), 4 (a good deal of your household waste), 3 (some of your household waste), 2 (few of your household waste), 1 (none of your household waste).

The Time 2 questionnaire recorded also respondents’ name and identification numbers to ensure that their responses at both time-points could be matched.

#### Participants and Procedure

The participants (overall *N* = 243) were students attending courses at the Faculty of Medicine and Psychology of Sapienza University of Rome (Italy). They were informed about the goals of the study and gave their informed oral consent to participate in it in exchange for course credits. Because the topic of the study and the procedure applied was not invasive, based on our institutional regulation, ethical committee approval was not requested. Participants’ responses were recorded at two time points, 1 month apart, during the academic year 2017–2018. Of the 243 questionnaires collected at time 1, 222 were considered complete and valid, thus final sample at Time 1 comprised 69 men and 153 women, and ranged in age 18–50 years (*M* = 21.26; *SD* = 3.97). Participants at Time 2 were the same as those who had participated at Time 1, but the overall questionnaires collected were inferior in number because only half (118) of the original respondents were present at that time (Mean age = 21.11; DS = 2.89; Males = 29, Females = 89).

#### Data Analysis

We decided to perform the analyses on two distinct samples: sample 1 (*N* = 222) including participants that had responded only to the questionnaire used at Time 1, and sample 2 (*N* = 118) including participants that had responded to the questionnaires both at Time 1 and Time 2. This decision was made due to the restricted number of participants that eventually responded to both questionnaires which would have limited the possibility to apply the statistical techniques we intended to use for testing H1 and H2. Hence, H1 and H2 were tested using sample 1, while H3 was tested using sample 2.

Data analysis started with the assessment of the construct validity and reliability of our measures. The measurement model of the TPB has undergone hundreds of tests in the literature and its validity can thus be considered as rather stable across situations and contexts. For this reason we performed a set of Confirmatory Factor Analyses, via SEM (Structural Equation Modeling) that had the main goal to confirm the distinction between the four measures of Local Norms (respectively referring to: inhabitants of the same city – LNC, inhabitants of the same area of the city – LNA, neighbors – LNN, and people living at the same place of respondents – LNP) and the measures of the other TPB components.

Subsequently, other two sets of statistical analyses were performed. The first set was performed on sample 1 (*N* = 222) and was directed to test the hypotheses (H1 and H2) concerning the predictors of behavioral intentions. In particular, these analyses were designed to assess whether the factors originally specified in the TPB had a statistically significant direct effect on behavioral intentions. We also compared the separate effects of the four local norms variables on behavioral intentions. This was done by performing a five steps hierarchical multiple regression with behavioral intention as the criterion variable. The first step represented the original TPB model, with Attitude, Subjective Norms, and PBC as predictors. The subsequent four steps added LNC, LNA, LNN, and LNP, respectively.

The second set of analyses was performed on sample 2, i.e., on the data from participants (*N* = 118) who had provided valid responses to both questionnaires (at Time 1 and Time 2). The aim was to test the thesis that local norms had a direct effect on self reported behavior, over and above the expected effects of all the other components of the TPB, including behavioral intentions. To this aim, a three steps hierarchical multiple regression was performed which tested for the effects of attitude, subjective norms, PBC (step1), intentions (step 2), and local norms (step 3) on the criterion variable (self-reported behavior).

Because responses provided to the variables considered in our study did not vary significantly in function of age or gender, these factors were not taken into account in the analyses performed.

#### Items Aggregations and Test of the Measurement Model

Negatively or inversely oriented indicators were reversed in score before starting the analyses. Subsequently, an exploratory factor analysis (EFA) based on principal axis factoring (PFA) was performed on all the indicators and used as a basis for the parceling process (i.e., the overall reduction in number of observed variables through aggregation). This was done to avoid computational problems and obtain smaller standard errors in the subsequent statistical analyses, and led to the construction of two new aggregated indicators per each construct investigated. In particular, for what concerns the attitude construct, ATT1 was computed by averaging participants’ responses to the items appropriate/inappropriate, pleasant/unpleasant, useful/useless, ATT2 (good/bad, right/wrong, and important/non-important); for PBC1 (difficult/easy, simple/complicated), PBC2 (possible/impossible, feasible/unfeasible). For subjective and local norms aggregations were made by separately averaging the scores of the indicators of their prescriptive and descriptive components. Finally, for intentions and self-reported behavior the indicators used in the analyses corresponded to those observed.

The new “observed” indicators were then used for testing the two measurement models via SEM with latent variables, using data sets from sample 1 (CFA1,2,3,4,5) and sample 2 (CFA 6). All models tested showed good fit (see Table 1). Descriptive statistics for the variables measured, internal coherence and bivariate correlations among the indicators tested on sample 1 (CFA1,2,3,4, and 5) and sample 2 (CFA 6) are reported in Tables 2, 3, respectively. Descriptive statistics, internal coherence and bivariate correlations between the latent variables for the two samples are displayed in Tables 4, 5. Some clarifications must be provided regarding the model tested on sample 2 (CFA6). Indications regarding the minimum sample size for obtaining unbiased estimates or standard errors with CFA based on SEM are quite variable in the literature suggesting that no universally valid rule can be defined. While a sample size larger than 150 (or even 200) is recommended in most cases, results of simulations studies indicate that with at least 100 subjects in a sample it is already possible to reduce the risks mentioned to 5% of the cases (Anderson and Gerbing, 1984). These suggestions are based on the ratio of cases to free parameters, but there may be other important indicators to consider, like, for example, the average magnitude of the loadings between the construct and the latent dimension (i.e., standardized structural coefficients) which should be at least 0.60 (e.g., Jackson, 2007). This condition was respected by the majority of the indicators analyzed with sample 2. Most important, for all the measures used in our study, results are coherent with those obtained in previous investigations. This indicates that despite the possible violation of some “rule of thumb” existing in the literature, the likelihood that we are committing a type 1 error (i.e., accepting an invalid measurement model) could reasonably be considered low.

**Table 1:** Results of the six CFAs assessing the validity of the measures used in all the models tested in the study.

Model	χ^2^_(df)_	*p*=	RMSEA	SRMR	CFI	NNFI
TPB + LNC^∗^	34.49_(27)_	0.152	0.035	0.055	0.99	0.98
TPB + LNA^∗^	27.98_(25)_	0.309	0.023	0.030	0.99	0.99
TPB + LNN^∗^	32.15_(25)_	0.154	0.036	0.033	0.99	0.98
TPB + LNP^∗^	42.22_(25)_	0.017	0.056	0.040	0.98	0.96
TPB + LN^∗∗^	26.55_(26)_	0.433	0.01	0.067	1.00	1.00


*^∗^The CFA tested the validity of measures of the all original constructs of the TPB, plus the specific type of Local Norm indicated (either C, city; A, city area; N, neighborhood; P, place of residence), on the data collected at Time 1, *N* = 222. ^∗∗^The CFA tested the validity of the measures of the original TPB comprising self-reported behavior plus an overall measure of LN (which aggregates LNC, LNA, LNN, and LNP), on the data set including only those participants who responded to both Time 1 and Time 2 questionnaires, *N* = 118.*

**Table 2:** Means (*M*), standard deviations (*SD*), standardized structural coefficients (λ), and Pearson’s correlations for measures of recycling attitudes (ATT), subjective norms (SN), perceived behavioral control (PBC), local norms (LN), and intentions (INT) used in the test of the measurement model on sample 1 (*N* = 222).

	*M*	*SD*	λ^	1	2	3	4	5	6	7	8	9	10	11	12	13	14	15	16
1. ATT1	4.97	0.80	0.91	1	0.68**	0.34**	0.40**	0.41**	0.50**	0.09	0.12	0.23**	0.13*	0.27**	0.12	0.28**	0.26**	0.24**	0.45**
2. ATT2	5.58	0.68	0.75		1	0.34**	0.28**	0.30**	0.43**	0.07	0.12	0.17*	0.18**	0.23**	0.16*	0.26**	0.31**	0.25**	0.38**
3. NSPR	4.66	1.07	0.76			1	0.52**	0.17*	0.22**	0.24**	0.23**	0.38**	0.24**	0.39**	0.24**	0.39**	0.35**	0.30**	0.43**
4. NSDS	4.18	1.21	0.67				1	0.29**	0.25**	0.35**	0.22**	0.49**	0.26**	0.50**	0.27**	0.50**	0.37**	0.34**	0.41**
5. PBC1	3.90	1.29	0.50					1	0.31**	0.22**	0.02	0.19**	0.06	0.15*	0.03	0.17*	0.07	0.15*	0.28**
6. PBC2	5.40	0.89	0.62						1	0.08	0.12	0.23**	0.12	0.25**	0.09	0.28**	0.16*	0.18**	0.27**
7. LNC1	3.63	1.10	0.83.							1	0.41**	0.61**	0.42**	0.44**	0.31**	0.36**	0.19**	0.19**	0.27**
8. LNC2	4.86	1.27	0.49								1	0.36**	0.90**	0.31**	0.78**	0.24**	0.58**	0.14*	0.19**
9. LNA1	4.02	1.27	0.96									1	0.46**	0.87**	0.46**	0.72**	0.33**	0.45**	0.45**
10. LNA2	4.96	1.22	0.48										1	0.40**	0.88**	0.31**	0.63**	0.19**	0.23**
11. LNN1	4.43	1.40	0.94											1	0.45**	0.93**	0.46**	0.54**	0.51**
12. LNN2	5.05	1.19	0.48												1	0.33**	0.67**	0.22**	0.28**
13. LNP1	4.72	1.50	0.90													1	0.50**	0.53**	0.54**
14. LNP2	5.32	1.17	0.55														1	0.24**	0.33**
15. INT1	4.78	1.56	0.61															1	0.48**
16. INT2	4.76	1.44	0.78																1


*p < 0.05; **p < 0.001; ^λ values for the indicators of attitudes, subjective norms, PBC, and intentions are average values across the 4 CFAs performed; all λ values are statistically significant; LN, local norms (C, city; A, city area; N, neighborhood; P, place of residence).

**Table 3:** Means (*M*), standard deviations (*SD*), standardized structural coefficients (λ), and Pearson’s correlations for measures of recycling attitudes (ATT), subjective norms (SN), PBC, local n*o*rms (LN), intentions (INT), and self-reported behavior (SRB), used in the test of the measurement model on sample 2 (*N* = 118).

	*M*	*SD*	λ^	1	2	3	4	5	6	7	8	9	10	11	12
1. ATT1	5.03	0.78	0.86	1	0.66**	0.36**	0.39**	0.38**	0.25**	0.18	0.08	0.18	0.40**	0.37**	0.39**
2. ATT2	5.68	0.51	0.77		1	0.32**	0.31**	0.28**	0.14	0.20*	0.07	0.29**	0.37**	0.35**	0.40**
3. SNPR	4.75	1.05	0.66			1	0.50**	0.17	0.16	0.42**	0.21**	0.36**	0.43**	0.44**	0.46**
4. SNDS	4.30	1.24	0.71				1	0.24**	0.26**	0.50**	0.27**	0.33**	0.38**	0.40**	0.37**
5. PBC1	4.04	1.35	–					1	0.75**	0.18	0.02	0.13	0.31**	0.25**	0.30**
6. PBC2	3.87	1.39	–						1	0.14	–0.04	0.15	0.28**	0.21*	0.28**
7. LN1	3.96	1.07	0.96							1	0.46**	0.48**	0.43**	0.58**	0.55**
8. LN2	4.84	1.19	0.48								1	0.25**	0.25**	0.24**	0.25**
9.INT1	4.78	1.58	0.61									1	0.40**	0.56**	0.57**
10. INT2	4.84	1.39	0.62										1	0.52**	0.53**
11. SRB1	4.81	1.35	0.95											1	0.91**
12. SRB2	4.60	1.29	0.95												1


*p < 0.05; **p < 0.001; SNPR, prescriptive subjective norms; SNDR, descriptive subjective norms; LN1, descriptive local norms; LN2, prescriptive local norms; all λ values are statistically significant; λ values for PBC have not been reported as this factor was excluded from the compute of the final model.

**Table 4:** Means (*M*), standard deviations (*SD*), reliability coefficients and bivariate correlations among the overall measures of attitudes (ATT), subjective norms (SN), PBC, local norms (LNC, LNA, LNN, and LNP), and intentions (INT), computed on sample 1 (*N* = 222).

	*M*	*SD*	1	2	3	4	5	6	7	8
1. ATT	5.28	0.68	*0.81*	0.43**	0.48**	0.12	0.19**	0.25**	0.33**	0.41**
2. SN	4.42	0.99		*0.68*	0.32**	0.37**	0.47**	0.49**	0.55**	0.50**
3. PBC	4.08	0.48			*0.45*	0.19**	0.22**	0.17*	0.26**	0.29**
4. LNC	3.88	1.01				*0.58*	0.77**	0.55**	0.41**	0.29**
5. LNA	4.15	1.09					*0.61*	0.80**	0.62**	0.45**
6. LNN	4.34	1.27						*0.63*	0.73**	0.55**
7. LNP	4.84	1.30							*0.65*	0.62**
8. INT	4.77	1.29								*0.65*


*p < 0.05; ** *p* < 0.001; Cronbach’s alpha coefficients are reported in the diagonal.

**Table 5:** Means (*M*), standard deviations (*SD*), reliabilitycoefficients and bivariate correlations among the overall measures of attitudes (ATT), subjective norms (SN), PBC, overall local norms (LN), intentions (INT), and self-reported behavior (SRBEHAV), computed on sample 2 (*N* = 118).

	*M*	*SD*	1	2	3	4	5	6
1. ATT	5.35	0.59	*0.75*	0.45**	0.32**	0.27**	0.39**	0.42**
2. SN	4.52	0.99		*0.66*	0.26**	0.57**	0.51**	0.49**
3. PBC	3.96	1.28			*0.86*	0.14	0.27**	0.28**
4. LN	4.42	0.96				*0.83*	0.59**	0.64**
5. INT	4.81	1.25					*0.57*	0.67**
6. SRBEHAV	4.71	1.30						*0.95*


*p < 0.05; ** *p* < 0.001; Cronbach’s alpha coefficients are reported in the diagonal. PBC has been eventually excluded from the analyses.

## Results

### Testing the Effects of the Four Types of Local Norms on Behavioral Intentions

Once validity and reliability of our measures had been assessed, aggregated scores were computed (by averaging the corresponding responses) to obtain an overall “observed” variable per each construct investigated. Hence, to test for H1 and H2, seven aggregated variables were constructed and used as indicators of, respectively, attitude, subjective norms, PBC, LNC, LNA, LNN, and LNP. To test for H3, LNC, LNA, LNN, and LNP were further aggregated into a single, overall Local Norm variable.

Table 6 shows results of the hierarchical regression analysis performed on data from sample 1 (*N* = 222). Together the original TPB variables accounted for a significant proportion of variance in behavioral intentions (*R*^2^ = 0.29), although only attitudes and subjective norms were statistically significant predictors of the criterion; PBC effects on intentions resulted as non-significant. The subsequent steps of the regression analyses confirmed that local norms can be considered adjunctive predictors of intention to recycle household waste. Each one of the four measures of local norms accounted for additional variance in intentions after taking into account the effects of the original TPB components, although only LNN and LNP remained significant when they were introduced together in the analyses. This result, however, confirms our hypothesis that local norms are adjunctive predictors of behavioral intentions and that their effects on intentions can vary as a function of the spatial proximity to a person’s place of residence. In particular, we found that, in our case, the closer the social category from which a local norm was derived was, physically, to a participant’s place of residence, the stronger was the predictive capacity of that norm *vis a vis* the intention to recycle household waste. Moreover, local norms effects overlapped only partially with those of people important to the subject (i.e., subjective norms); although the effect of subjective norms on intentions decreased substantially when local norms were introduced to the model, it nevertheless remained statistically significant. In other words, although local norms and subjective norms may overlap to some extent, they also seem able to tap different aspects of normative influence.

**Table 6:** Hierarchical regression analysis predicting Intentions to participate in household waste recycling on sample 1 (*N* = 222).

	β coefficients					Adjusted *R*^2^	*R*^2^ change
	***Step1***	***Step 2***	***Step 3***	***Step 4***	***Step 5***		
ATT	0.22**	0.23**	0.23**	0.20**	0.18**	0.29	
SN	0.38**	0.33**	0.26**	0.21**	0.13*		
PBC	0.06	0.05	0.04	0.07	0.05		
LNC		0.13*	–0.13	–.07	–0.04	0.30	0.01
LNA			0.37**	0.03	–0.03	0.35	0.05
LNN				0.40**	0.21*	0.40	0.05
LNP					0.36**	0.45	0.05


*p = <0.05; **p = <0.01; ATT, attitude; SN, subjective norms; PBC, perceived behavioral control; LN, local norms (C, city; A, city area; N, Neighborhood; P, place of residence).

### Test of the Effects of the Overall Local Norms on Self-Reported Behavior

Table 7 shows results of the hierarchical regression analysis performed on data from sample 2 (*N* = 118). Consistently with the model’s tenets, intentions resulted as the main predictor of self-reported behavior. However, coherently with our H3, Local Norms were able to explain an additional proportion of variance of the criterion, over and above that explained by the original components of the model considered together (see Table 7).

**Table 7:** Hierarchical regression analysis predicting self-reported participation in household waste recycling on sample 2 (*N* = 118).

	*Step1*	*Step 2*	*Step 3*	Adjusted *R*^2^	*R*^2^ change
ATT	0.22**	0.13	0.16*	0.28	
SN	0.36**	0.14	0.06		
PBC	0.12	0.06	0.07		
INT		0.52**	0.43**	0.47	0.19**
LN			0.24**	0.51	0.04**


*p = <0.05; **p = <0.01; ATT, attitude; SN, subjective norms; PBC, perceived behavioral control; INT, intentions; LN, local norms (overall measure).

## Discussion and Conclusion

The goal of the study reported in the present paper was to deepen some aspects of the effects of local norms on behavioral intentions within the TPB framework. The results support previous findings as they confirm that adding a local norms variable to this model explains significant additional variance in behavioral intentions (Carrus et al., 2009; Fornara et al., 2011). Moreover, they add to the existing literature as they provide evidence that the effects of local norms varies as a function of the spatial proximity (to the individual) of the social category relevant to a certain behavior, in a given social-physical context. In the particular case we investigated, that of household waste recycling, the normative influence of neighbors and people living at the same address of respondents (housemates) appeared to be of considerable relevance in shaping behavioral intentions. Respondents also appeared to be sensitive to the normative influence of people living in the same area of the city, or simply in the same city, although the effects of these factors tended to be eventually obscured in the analyses by those from more proximal types of local norms (i.e., referred to neighbors and housemates).

It should be noted, then, that our study provides further evidence to the idea that the effect of local norms on respondents’ intentions differs substantially from the effects of norms derived from people with whom an individual has affective bonds (like for e.g., family members, close friends, and partners), who may also live nearby. The latter type of normative influence, which is of paramount importance in people’s life, seems to be well captured by the subjective norms construct included in the TPB, and it may depend on a number of psychological processes likely activated by close relationships (affection, interdependence, identification, etc.; for alternative explanations see e.g., Vesely and Klöckner, 2018). The additional influence of local norms on behavioral intentions (within the TPB), instead, derives from different psychological and psychosocial processes, probably linked to the individual’s awareness of the implications of sharing a certain social-physical environment with a number of other people (e.g., Granovetter, 1973). In this context, certain behaviors assume specific collective meanings and implications which add to the meanings and implications that the same behavior may have in the context of one’s close friends and family. Further studies are needed in order to clarify the psychological processes underpinning local norms in general, but some hypotheses can be derived about their contribution to the TPB from the classical literature on social influence and on social dilemmas. For example, this literature suggests that people may conform to the behavior of others even when these consist of strangers or people only generically known and even when they are physically absent from the context in which the behavior occurs (provided their possible behavior can be inferred from cues existing in the environment; e.g., Chartrand and Bargh, 1999; Cialdini and Goldstein, 2004; Cialdini, 2005, 2007; Bargh et al., 2012). Because this inclination to conformity is deemed to be rather automatic, the hypotheses put forward to explain it encompass the possibility of an informative/adaptive purpose. It is posited that, by observing others’ behavior people can rapidly and (effortlessly) learn the apparently wisest (and socially approved) thing to do in that context. This is empirically supported by those studies that showed how the effects of descriptive norms on behavioral intention tend to increase with the lowering of the cognitive level of elaboration (e.g., Melnyk et al., 2011; Kredentser et al., 2012). As a matter of fact, Fornara et al. (2011) showed that the additional contribution of local norms to the TPB is mostly linked to its descriptive component.

Moreover, conformity offers the opportunity to share responsibility for misbehaviors, which could be readily justified using arguments such as “everyone else was doing the same thing” (e.g., Chen and Gifford, 2015). Literature on social dilemmas has extended this account, suggesting additional explanations relevant to behaviors directed at goals that can only be achieved through collective, coordinated effort (e.g., Van Lange et al., 2013). In these cases, the informative power of local norms may also derive from the possibility that they offer to understand whether the individual contribution is actually worthwhile. Ecological behaviors tend to be rather costly at personal level, whereas the beneficial effects are uncertain and postponed to an undetermined collective future. Hence, people may be unwilling to behave in a way that contributes to the attainment of common goals in the future if they perceive that others are not doing likewise, here and now (see Fornara et al., 2011). Taken together, these hypotheses can also explain the direct effect (over and above that of intentions) of local norms on self-reported behavior that we observed. Behavioral intentions (in the TPB) account for the effects of reasoned processes based on the conscious evaluations of attitudinal, normative and control related pros and cons of a behavior. Although it is plausible that part of the influence of local norms may derive from similar conscious processes, it is also likely that their additional effect on self-reported behavior in the TPB is due to the automatic component discussed above, which might be sufficient on its own, to induce a person to conform to others’ behavior in a certain social-physical context and time. The effects of local norms on behaviors that we observed indicate that, independently of an individual’s overt intention, the probability that someone will recycle household waste is directly affected by perceptions of the behavior of others in the local physical environment. In other words, local norms might be able to seize the effects of some heuristic/peripheral processes that are not taken into account by the central reasoned/planned factors constituting this model (see also Fornara et al., 2011).

Our study also adds to the literature on ecological behaviors in general and waste recycling in particular. Recycling is becoming common practice in many countries, as many local governments have encouraged it by providing the necessary infrastructures and facilities (e.g., Thomas and Sharp, 2013). Citizens’ attitudes have become more favorable and thus the problem of increasing recycling rates and accuracy seems today more linked to a deeper understanding of its social and collective implications (see also Farrow et al., 2017). Our results provide further evidence of the importance of considering the role of different types of normative influence, which may act at different scales of proximity to the individual and may be driven by different social-psychological processes. For example, many authors have already acknowledged how, in addition to its implications for the single individual, household waste recycling, may have different meanings at the familial, interpersonal (e.g., Oates and McDonald, 2006; Grønhøj and Thøgersen, 2012), and group level (e.g., White et al., 2009). Furthermore, the normative influences to which an individual is subject with respect to recycling may vary during the life span. This implies that studies collecting data that will be used to plan recycling initiatives should pay more attention to the particular characteristics of the social-physical context in which the initiative will be implemented, as well as to how they may change in the future.

### Limits of the Study

This study has some limitations that need to be considered.

- Our decision to focus the study on university students was motivated by the possibility to include a higher number of people who did not live with parents, relatives or partners. However, this aspect was not measured in the questionnaire we used in our study. It could be included in future studies in order to replicate our investigation on more heterogeneous samples of the population.
- Previous research work by Fornara et al. (2011) have provided empirical evidences and discussed the differential contribution of prescriptive and descriptive norms (referred to both subjective norms and local norms) to the TPB. This distinction was not considered in this study and thus represents another aspect that should be further explored in the future.
- The sample size of respondents at time 2 only reaches the minimum requested for the application of the statistical techniques (Structural Equation Modeling – SEM) used to evaluate the validity of the measurement model. Although alternative statistical techniques that we have performed (i.e., principal axis factoring – PFA) confirm the results obtained with the SEM procedures, still our findings should be considered of preliminary nature and need to be confirmed by studies conducted on larger samples and in different contexts.
- The proportion of variance explained by the model is lower for intentions than for self-reported behavior, and it is also lower than the proportion of variance explained in other studies that have applied a similar questionnaire (Carrus et al., 2008; Fornara et al., 2011). This could be due to the non-significant effect of PBC (which explained an important proportion of variance in previous studies), or to some crucial factors relevant for explaining intentions which could be missing from this model. For example, given the young age of our respondents it is possible that we did not include all the normative influence relevant to people of this age (like for example those from important peers’ groups).
- Perceived behavioral control has often played a key role in the prediction of pro-environmental behavioral intentions, whereas its contribution to the TPB was non-significant in the present case. One of the reasons could be the low level of reliability of the corresponding measure, which might have weakened its overall power in the prediction of intentions (some of the local norms indicators showed a similar pattern of low reliability and a weak contribution to the prediction of intentions as well). However, there might be other explanations as well, like the particular characteristics of our sample, mostly composed of young university students. Some previous studies have shown that recycling skills (a factor strictly linked to perceived control; e.g., Passafaro and Livi, 2017) may, in some cases, increase with respondents’ level of education (e.g., Passafaro et al., 2016). In addition, more simply, it is possible that personal attitudes and social norms tend to represent the strongest motivating factors for behavior in this particular population. Indeed, it seems not so unreasonable to think that students’ decisions to recycle could actually be more influenced by social norms and personal attitudes rather than by issues related to the feasibility of the considered behavior (which, in the end, should not appear as particularly problematic to perform to highly educated people). This is another reason for replicating this study on larger and more heterogeneous samples of the population.

## Data Availability

The datasets generated for this study are available on request to the corresponding author.

## Author Contributions

This article, and the study described in it, was conceived and carried out by all the authors conjunctly. SL and AK conducted the data collection. PP and SL provided a major contribution to the data analysis and preparation of the manuscript. AK provided some constructive feedbacks and contributed to the preparation of some sections of the manuscript.

## Conflict of Interest Statement

The authors declare that the research was conducted in the absence of any commercial or financial relationships that could be construed as a potential conflict of interest.

## Footnotes

1 http://people.umass.edu/aizen/pdf/tpb.measurement.pdf
